# Supplementary material for: Ancestral allele of DNA polymerase gamma modifies antiviral tolerance
Source: Nature. 2024 Apr 3;628(8009):844–53. doi: 10.1038/s41586-024-07260-z (PMC11041766; doi:10.1038/s41586-024-07260-z)
Supplement: Supplementary file 1 — Uncropped immunoblots from Figs. 1–3 and 5 and Extended Data Figs. 1–7. [file 41586_2024_7260_MOESM1_ESM.pdf]

nature portfolio

https://doi.org/10.1038/s41586-024-07260-z

Supplementary information

Ancestral allele of DNA polymerase gamma modifies antiviral tolerance

In the format provided by the
authors and unedited

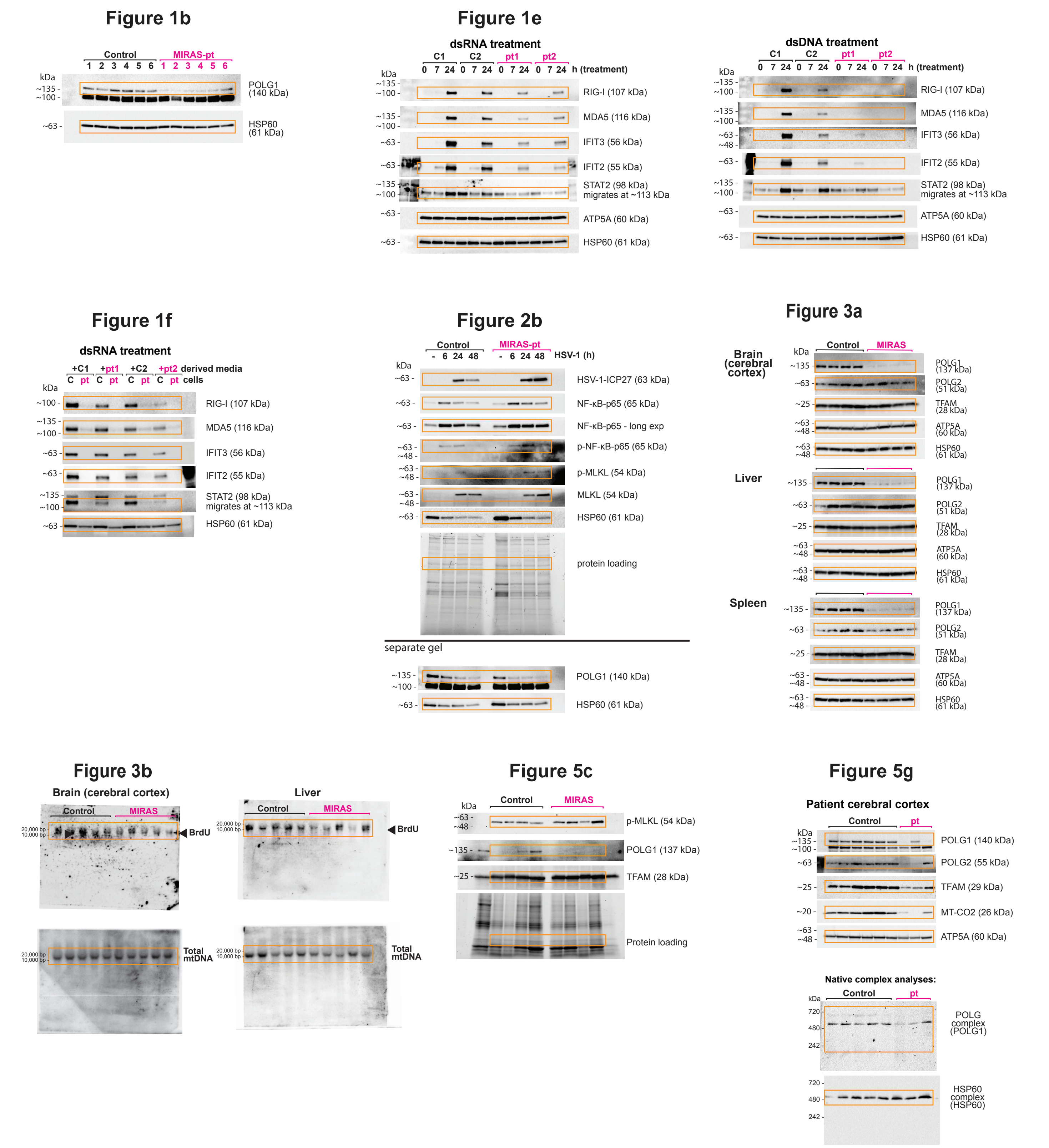

Supplementary Figure 1

Extended Data Figure 1d

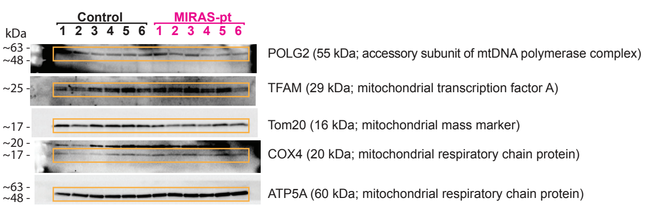

Extended Data Figure 2b

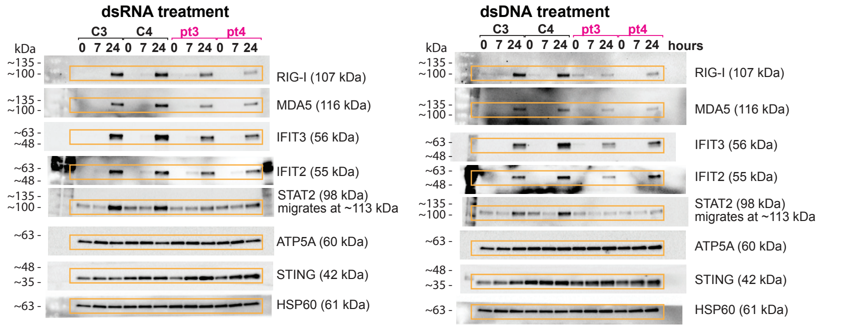

Extended Data Figure 2c

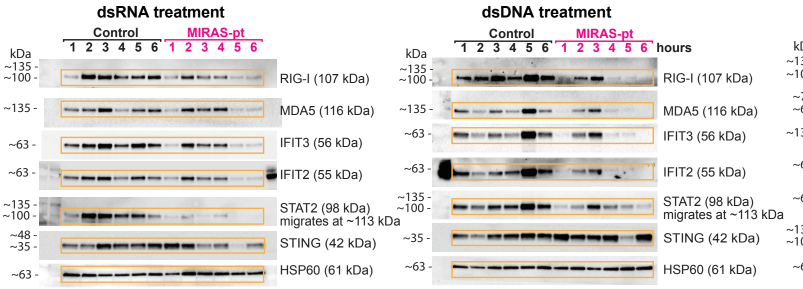

Extended Data Figure 3a

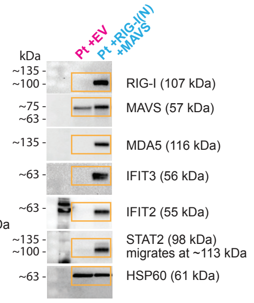

Extended Data Figure 3c

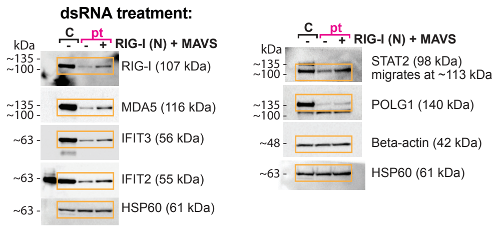

Extended Data Figure 3e

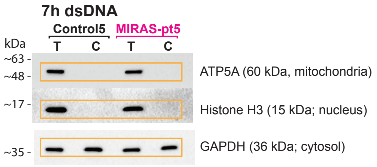

Extended Data Figure 3g

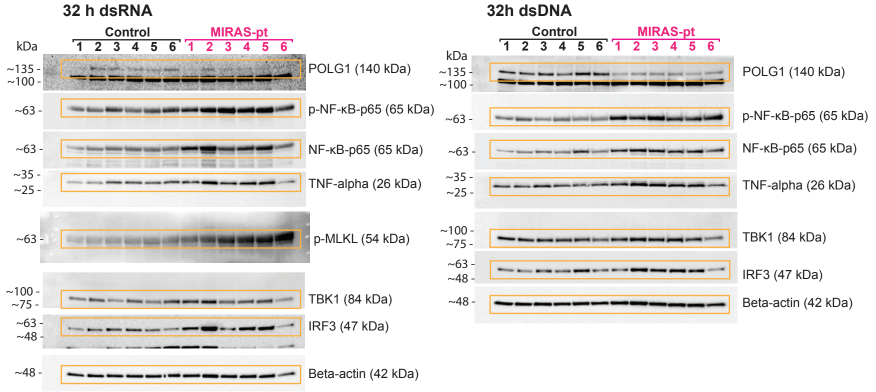

Extended Data Figure 3i

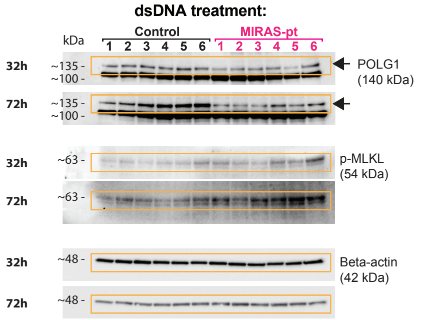

Supplementary Figure 1

Extended Data Figure 4a

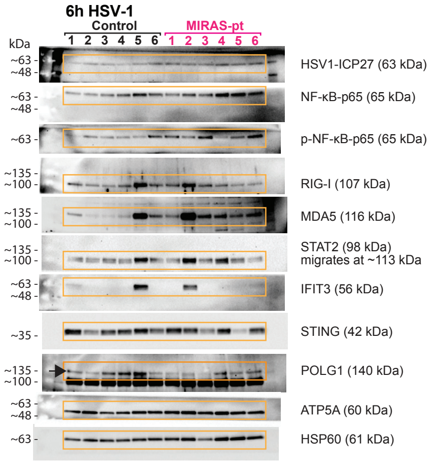

Extended Data Figure 4b

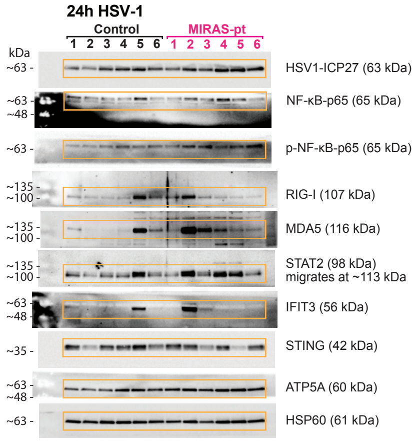

Extended Data Figure 4c

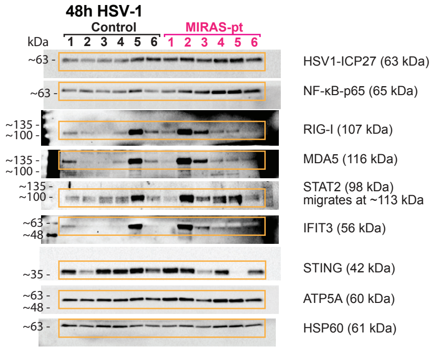

Extended Data Figure 4e

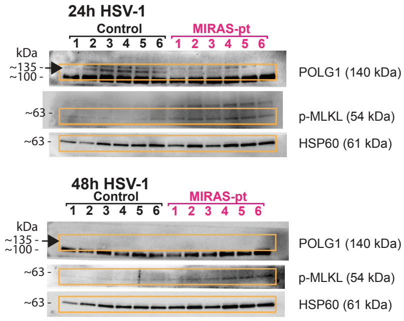

Extended Data Figure 5b

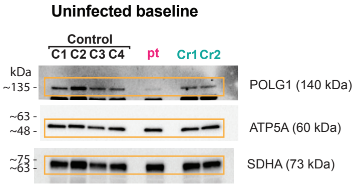

Extended Data Figure 5c

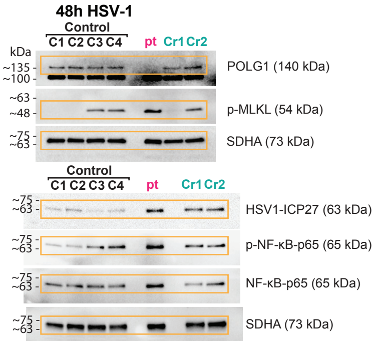

Extended Data Figure 5e

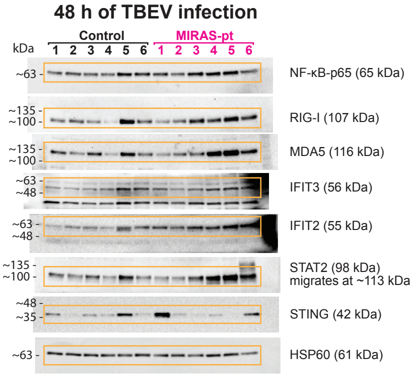

Extended Data Figure 5f

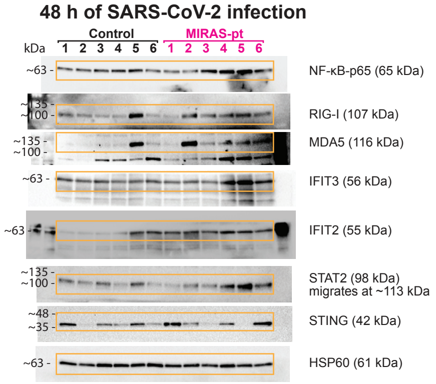

Extended Data Figure 5g

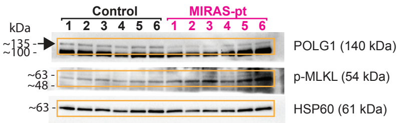

Extended Data Figure 5h

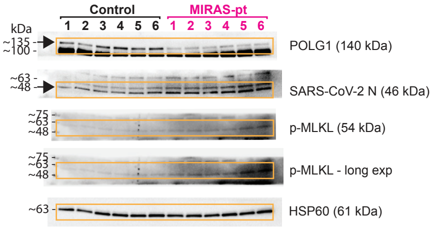

Extended Data Figure 6c

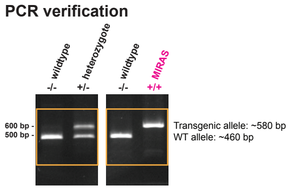

Extended Data Figure 7b

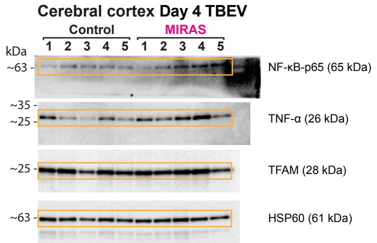

Extended Data Figure 8a

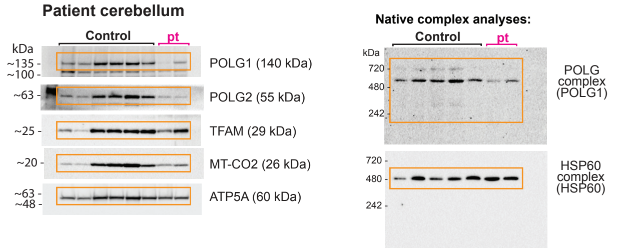

Supplementary Figure 1
