# Supplementary material for: Ancestral allele of DNA polymerase gamma modifies antiviral tolerance
Source: Nature. 2024 Apr 3;628(8009):844–53. doi: 10.1038/s41586-024-07260-z (PMC11041766; doi:10.1038/s41586-024-07260-z)
Supplement: Supplementary file 2 — Reporting Summary [file 41586_2024_7260_MOESM2_ESM.pdf]

nature portfolio

Corresponding author(s): Anu Suomalainen

Last updated by author(s): Jan 30, 2024

Reporting Summary

Nature Portfolio wishes to improve the reproducibility of the work that we publish. This form provides structure for consistency and transparency in reporting. For further information on Nature Portfolio policies, see our Editorial Policies and the Editorial Policy Checklist.

Statistics

For all statistical analyses, confirm that the following items are present in the figure legend, table legend, main text, or Methods section.

n/a	Confirmed	
☐	☒	The exact sample size (*n*) for each experimental group/condition, given as a discrete number and unit of measurement
☐	☒	A statement on whether measurements were taken from distinct samples or whether the same sample was measured repeatedly
☐	☒	The statistical test(s) used AND whether they are one- or two-sided<br>*Only common tests should be described solely by name; describe more complex techniques in the Methods section.*
☐	☒	A description of all covariates tested
☐	☒	A description of any assumptions or corrections, such as tests of normality and adjustment for multiple comparisons
☐	☒	A full description of the statistical parameters including central tendency (e.g. means) or other basic estimates (e.g. regression coefficient) AND variation (e.g. standard deviation) or associated estimates of uncertainty (e.g. confidence intervals)
☐	☒	For null hypothesis testing, the test statistic (e.g. F, t, r) with confidence intervals, effect sizes, degrees of freedom and P value noted<br>*Give P values as exact values whenever suitable.*
☒	☐	For Bayesian analysis, information on the choice of priors and Markov chain Monte Carlo settings
☒	☐	For hierarchical and complex designs, identification of the appropriate level for tests and full reporting of outcomes
☒	☐	Estimates of effect sizes (e.g. Cohen's *d*, Pearson's *r*), indicating how they were calculated

Our web collection on statistics for biologists contains articles on many of the points above.

Software and code

Policy information about availability of computer code

Data collection	Fluorescence images were acquired using Zeiss AxioImager epifluorescent microscope<br>Histological images were collected using a light microscope equipped with a ProgRes® CCD Routine Camera (C7, JENOPTIK Optical Systems GmbH) with ProgRes® Capture Pro Camera Control Software 2.10.0.1.<br>Untargeted metabolite profiling was performed using flow injection analysis on an Agilent 6550 QTOF instrument (Agilent, Santa Clara, CA).<br>Proteomics data was collected by LC-MS/MS using an Easy nLC1000 liquid chromatography system (Thermo Electron, Bremen, Germany) coupled to a QExactive HF Hybrid Quadrupole-Orbitrap mass spectrometer (Thermo Electron) with a nanoelectrospray ion source (EasySpray, Thermo Electron).<br>For RNA sequencing analysis, the quality of the sequencing libraries was assessed using the TapeStation DNA High Sensitivity Assay (Agilent). The libraries were sequenced on a Illumina NextSeq 500.<br>Immunoblot and southern blot images were obtained using ChemiDoc™ XRS+ imaging machine (BioRad) and signals were quantified using Image Lab Version 6.1.0 build 7 software (BioRad).<br>Radioactive signals were developed using Typhoon™FLA7000 (GE Healthcare).<br>qPCR were performed using CFX96 or 384 Touch Real-Time PCR Detection System (BioRad).<br>ELISA assay was measured using Enspire Multimode plate reader (Perkin Elmer; Software version 4.13.3005.1482).
Data analysis	For proteomic analyses, the Uniprot human database (September 2018) was used was used for the database searches. Protein identification and label-free quantification was done using MaxQuant (ver 1.6.7.0) software followed by data filtering and statistical analysis with Perseus (ver 1.6.1.3) software.<br>For RNA seq, Read alignments were done using GRCm38 (mouse) reference genome and GENCODE Mouse Release 28 or GRCh38 (human) reference genome and GENCODE Human Release 33 comprehensive gene annotation files. Differential expression analysis was done in the DESeq2 software (as per default setting) in R environment.

For metabolomic data analysis, HMDB v4.0 and 3.0 database were used.
For functional/pathway enrichment analyses, Qiagen Ingenuity Pathway Analyses (QIAGEN Inc., https://digitalinsights.qiagen.com/IPA), g:Profiler (https://biit.cs.ut.ee/gprofiler) toolset and KEGG database were utilized for the analyses of transcriptome, metabolome and/or proteome datasets. For immune pathway analyses, we further utilized the manually curated InnateDB database (https://www.innatedb.com/index.jsp). The server default test/algorithm was utilized to calculate the p value also described in the figure legend.
Statistical analyses were performed using Microsoft Excel version 16.80, GraphPad Prism version 10.1.1 for macOS (GraphPad Software, www.graphpad.com) or using the server default test as described above.
Quantification of immunofluorescence signal was performed using CellProfiler 4.2.6 software and for immunohistology, liver immune infiltration area was measured using ImageJ 2.0.0-rc-69/1.52n software (https://imagej.net/ij/) whereas liver ORO and CDs signal were quantified using CellProfiler 4.2.6 software after pixel classification by ilastik 1.3.3 software.
Data compiling and processing was performed using Microsoft Excel version 16.80 and Adobe Illustrator 26.2.1.
Images were created using Adobe Illustrator 26.2.1. or with BioRender.com

For manuscripts utilizing custom algorithms or software that are central to the research but not yet described in published literature, software must be made available to editors and reviewers. We strongly encourage code deposition in a community repository (e.g. GitHub). See the Nature Portfolio guidelines for submitting code & software for further information.

Data

Policy information about availability of data

All manuscripts must include a data availability statement. This statement should provide the following information, where applicable:

- Accession codes, unique identifiers, or web links for publicly available datasets
- A description of any restrictions on data availability
- For clinical datasets or third party data, please ensure that the statement adheres to our policy

The mouse RNA-seq data generated in this study have been deposited into NCBI's Gene Expression Omnibus (GEO) and are accessible through GEO series accession number GSE249432. Metabolomic data have been deposited to the MassIVE database and are accessible through MSV000093634. Human omics data sharing is restricted because of European general data protection regulations (GDPR) laws. Individual enquiry of expression changes of specific genes /proteins can be directed to the corresponding author. Numerical source data giving rise to graphical representation and statistical description in Figures 1-6 and Extended Data Figures 1-9 are provided as Source Data file and in Supplementary Tables 2-5. Characteristics of human research participants and materials used in this study are provided in Supplementary Tables 1 and 6 respectively. Uncropped images of southern/immunoblots presented in the figures are included in Supplementary Fig 1.

Research involving human participants, their data, or biological material

Policy information about studies with human participants or human data. See also policy information about sex, gender (identity/presentation), and sexual orientation and race, ethnicity and racism.

Reporting on sex and gender

We selected gender matched patient and controls in the fibroblast studies to control for potential immune variation due to gender induced effect. Similarly, for autopsy sample analyses, we used material derived from individuals of the same gender. The sex/gender of human participants isolated materials are described in the respective figure legends and in Supplementary Table 1.

Reporting on race, ethnicity, or other socially relevant groupings

The human participants are from Finnish population which is clinically relevant for the study of the MIRAS disease in this report which has high prevalence within Finnish population. No race, ethnicity or socially relevant groupings were applied.

Population characteristics

For population, see above. The human tissues samples were from autopsies, where the families had consented the materials for research use. The skin biopsies and blood samples were collected with consent from patients and control individuals. The characteristics of the human research participants including age, gender, genotype and diagnosis, are provided in Supplementary Table 1.

Recruitment

The blood and fibroblast samples were from patients who were previously diagnosed to carry the POLG1 mutation of interest and recruited and informed by their physician, who also collected the consents.

Ethics oversight

Human samples were collected and used with informed consents, according to the Helsinki Declaration and approved by the Ethical Review Board of Kuopio University Central Hospital (license number 410/2019). Patient and control materials included fibroblasts (established from skin biopsies from individuals' forearms), blood and autopsy-derived brain samples. Control samples were from voluntary healthy subjects (fibroblasts and sera) and for brains, from people who died acutely for non-CNS-disease causes. Autopsy sample collection was approved by the governmental office for social topics and health.

Note that full information on the approval of the study protocol must also be provided in the manuscript.

Field-specific reporting

Please select the one below that is the best fit for your research. If you are not sure, read the appropriate sections before making your selection.

☒ Life sciences ☐ Behavioural & social sciences ☐ Ecological, evolutionary & environmental sciences

For a reference copy of the document with all sections, see nature.com/documents/nr-reporting-summary-flat.pdf

Life sciences study design

All studies must disclose on these points even when the disclosure is negative.

Sample size	No sample size calculation were performed. For mice, sample sizes were chosen to ensure adequate power and to account for potential inter-individual/animal, gender and age variance (age and gender-matched samples were utilized as controls). The number of biologically independent mouse or different individual derived human samples was described in the respective figure legends. The limitation here was the virus exposure capacity in safety-level-3 facilities. For mouse analyses, we have at least 4 or more mice of the same sex per condition. For human samples (primary fibroblasts, autopsy-derived brain samples) the availability of patients limits the sample size. The experiments were performed on six biologically independent patient-derived primary fibroblast lines compared to six age and gender matched control individual fibroblast lines (or otherwise as indicated in the figure legends). To further confirm the causal role of MIRAS p.W748S, we CRISPR-corrected the mutation in an iPSC cell line of a biological patient and generated two independent clones of iPSC-induced fibroblast from the corrected line, and compared their response to four different control individual cell lines. For brain samples we have n=3 patient cerebral cortex (n=2 cerebellum) and n=15 patient blood samples and their age/gender-matched controls (n=5/6 for brain samples or 23 for blood samples, respectively). For mouse analyses, we have at least 4 or more mice of the same sex per condition. The genetic association analyses were generated from the genome and health data collected by the FinnGen study from 309,154 individuals from Finnish population.
Data exclusions	No human or mouse data point were excluded. Specific filtering criteria are included in our omic analyses as described in the method section.
Replication	Biologically replicates or independent human-derived samples (of matched gender and age) are included to ensure reproducibility: 6 independent human derived primary fibroblast lines that were isolated from 6 different control individuals or patients (or as indicated in the figure legends) and 4 or more mice were analyzed (exact number of mice analyzed per analyses are indicated in the figure legends); for human tissue samples - the number is limited by the availability of the material and the exact number of different individual derived samples is described in the respective figure legends. The cell experiments were replicated at least once using independent individual derived line(s). For virus infection, the experiment was tested with a few biological cell lines and then replicated for the full set of N=6 human cell lines (or as indicated in the respective figure legends). Where representative data are shown, the experimental findings were replicated in independent biological cell lines/mice (number as indicated in the respective figure legends) and quantification of the each biological replicates are included in the data representation.
Randomization	This is not relevant to our study with human participants as no conduct of research to obtain data or samples through intervention or interaction with individuals.<br>No specific method of randomization was used to select animals or human cell lines. Human cell lines and mice of targeted genotype were screened and selected to investigate the effect of the disease gene mutation in the phenotype and response to viral infection.
Blinding	This is not relevant to our study with human participants as no conduct of research to obtain data or samples through intervention or interaction with individuals.<br>No blinding was done with the mouse group generation as this was predetermined by the mouse genotype.<br>Histological evaluation of mouse GABAergic defect evaluation was performed blindly.<br>Mouse liver histological evaluation was not performed blindly but was independently scored by 2 researchers.

Reporting for specific materials, systems and methods

We require information from authors about some types of materials, experimental systems and methods used in many studies. Here, indicate whether each material, system or method listed is relevant to your study. If you are not sure if a list item applies to your research, read the appropriate section before selecting a response.

Materials & experimental systems

n/a	Involved in the study	
☐	☒	Antibodies
☐	☒	Eukaryotic cell lines
☒	☐	Palaeontology and archaeology
☐	☒	Animals and other organisms
☒	☐	Clinical data
☒	☐	Dual use research of concern
☒	☐	Plants

Methods

n/a	Involved in the study	
☒	☐	ChIP-seq
☒	☐	Flow cytometry
☒	☐	MRI-based neuroimaging

Antibodies

Antibodies used	All primary antibodies used were described below and in Extended data table 6,<br>Antibodies; Source; Catalogue number (dilution factor: refer to the Extended Data Table 6)<br>Antibodies Source Catalogue number<br>Anti-TOM20 Santa Cruz Biotechnology sc-11415<br>Anti-HSP60 Santa Cruz Biotechnology sc-1052<br>Anti-MDA5 Proteintech 21775-1-AP<br>Anti-STING Cell Signaling Technology 50494S<br>Anti-STAT2 Proteintech 16674-1-AP

Anti-RIG-I Cell Signaling Technology 3743S
Anti-TFAM Abcam Ab131607
Anti-POLG1 Abcam ab128899
Anti-POLG2 Proteintech 10997-2-AP
Anti-MT-CO2 Abcam ab110258
Anti-COX4 Abcam ab14744
Anti-IFIT3 Proteintech 15201-1-AP
Anti-IFIT2 Proteintech 12604-1-AP
Anti-Beta-Actin Cell Signalling Technology 3700S
Anti-Phospho-MLKL-T357/S358/S360 ABClonal AP0949
Anti-MLKL ABClonal A13451
Anti-ATP5A Abcam ab14748
Anti-Phospho-NF-kappaB p65 (Ser536) [93H1] Cell Signaling Technology 3033S
Anti-NF-kappaB p65 Santa Cruz Biotechnology sc-8008
Anti-TNF-alpha Abcam ab66579
Anti-IRF3 (D83B9) Cell Signaling Technology 4302S
Anti-TBK1/NAK (D1B4) Cell Signaling Technology 3504
Anti-CD68 [FA-11] Bio-Rad MCA1957
Anti-CD3 - mouse liver BD Biosciences 555273
Anti-CD4 BD Biosciences 550280
Anti-CD8b BD Biosciences 550797
Anti-CD45R [B220/RA3-6B2] BD Pharmingen MLDP7
Anti-CD3 [SP7] - mouse brain Spring Bioscience Corp., Ventana Medical Systems; Abcam ab16669
Anti-Ibal WAKO 019-19741
Anti-dsRNA Scicons 10010200
Anti-BrdU BD Pharmingen 555627
Anti-GAD67 [clone 1G10.2] Merck Millipore MAB5406
Anti-GABRB2 Abcam ab42598
Anti-GAPDH Cell Signaling Technology 2118S
Anti-Histone H3 Cell Signaling Technology 3638
Anti-HSV-1-ICP27 Santa Cruz Biotechnology sc-69806
Anti-SARS-CoV2-N Provided by Prof. Olli Vapalahti Department of Virology, Faculty of Medicine, University of Helsinki, Helsinki, Finland
Anti-TBEV Provided by Prof. Olli Vapalahti Department of Virology, Faculty of Medicine, University of Helsinki, Helsinki, Finland
Anti-TBEV (Hochosterwitz isolate) Donated by Prof. Karin Stiasny Center for Virology, Medical University of Vienna, Austria
Peroxidase AffiniPure™ Goat Anti-Mouse IgG (H+L) Jackson ImmunoResearch 115-035-146
Peroxidase AffiniPure™ Goat Anti-Rabbit IgG (H+L) Jackson ImmunoResearch 111-035-144
Donkey Anti-Goat IgG H&L (HRP) Abcam ab97110
ImmPRESS® HRP Goat Anti-Rat IgG, Mouse adsorbed Polymer Detection Kit, Peroxidase Vector Laboratories MP-7444-15
VECTASTAIN® Elite® ABC-HRP Kit, Peroxidase (Mouse IgG) Vector Laboratories PK-6102
VECTASTAIN® Elite® ABC-HRP Kit, Peroxidase (Rabbit IgG) Vector Laboratories PK-6101
EnVision+ anti-HRP, Rabbit Agilent Dako K4003
OmniMap anti-Rabbit HRP Ventana Medical Systems, Inc. 760-4311
Goat anti-Mouse IgG (H+L) Alexa fluor 594 Invitrogen A-11005
Goat anti-human IgG (H+L) Alexa fluor 488 Invitrogen A-11013

Validation

Antibodies were used according to the validation listed in manufacturer's instructions. The details of antibody validation are given in Supplementary Table 6.

Eukaryotic cell lines

Policy information about cell lines and Sex and Gender in Research

Cell line source(s)

Human skin biopsy-derived primary fibroblasts were generated in our lab from skin biopsies isolated from individuals' forearms.
Human neuroblastoma cells (SK-N-SH; https://www.atcc.org/products/htb-11), human non-small cell lung cancer cells (Calu-1; https://www.atcc.org/products/htb-54), and African green monkey kidney cells (Vero E6; https://www.atcc.org/products/crl-1586 and Vero; https://www.atcc.org/products/ccl-81) were purchased from ATCC company.

Authentication

All patient derived cell lines were verified to carry the POLG1 patient mutation of interest, validating them to represent the disease of interest.

Mycoplasma contamination

The cell lines have been tested for absence of mycoplasma contamination.

Commonly misidentified lines
(See ICLAC register)

n/a

Animals and other research organisms

Policy information about studies involving animals; ARRIVE guidelines recommended for reporting animal research, and Sex and Gender in Research

Laboratory animals

Mus musculus, C57BL/6JOlaHsd mice.
Mouse samples were collected from female mice age ~3 months, 1 and 2+ years old as indicated in the respective figure legends.

	Mice were housed in controlled room at 22°C (30-40% humidity) with 12h light/dark cycle and ad libitum access to food and water and were regularly monitored for weight and food consumption.
Wild animals	The study did not involve wild animals
Reporting on sex	We have used female mice in this study (except for behavioral analyses and in GABAergic marker staining where male and female mice were included but they were analysed separately based on the animal sex). The sex of the mice were stated in the respective figure legends.
Field-collected samples	The study did not involve samples collected from the field
Ethics oversight	Animal experimental procedures were approved by the Animal Experimental Board of Finland (license numbers ESAVI/689/4.10.07/2015 and ESAVI/3686/2021).

Note that full information on the approval of the study protocol must also be provided in the manuscript.

Plants

Seed stocks	*Report on the source of all seed stocks or other plant material used. If applicable, state the seed stock centre and catalogue number. If plant specimens were collected from the field, describe the collection location, date and sampling procedures.*
Novel plant genotypes	*Describe the methods by which all novel plant genotypes were produced. This includes those generated by transgenic approaches, gene editing, chemical/radiation-based mutagenesis and hybridization. For transgenic lines, describe the transformation method, the number of independent lines analyzed and the generation upon which experiments were performed. For gene-edited lines, describe the editor used, the endogenous sequence targeted for editing, the targeting guide RNA sequence (if applicable) and how the editor was applied.*
Authentication	*Describe any authentication procedures for each seed stock used or novel genotype generated. Describe any experiments used to assess the effect of a mutation and, where applicable, how potential secondary effects (e.g. second site T-DNA insertions, mosiacism, off-target gene editing) were examined.*
